# Trajectories of quality of life in breast cancer survivors during the first year after treatment: a longitudinal study

**DOI:** 10.1186/s12905-022-02153-7

**Published:** 2023-01-10

**Authors:** Jin-Hee Park, Yong Sik Jung, Ji Young Kim, Sun Hyoung Bae

**Affiliations:** 1 College of Nursing, Research Institute of Nursing Science, Ajou University, 164, World cup-ro, Yeongtong-gu, Suwon, 16499 Korea; 2 Department of Breast Surgery, School of Medicine, Ajou University, Suwon, Korea

**Keywords:** Breast neoplasms, Cancer survivors, Longitudinal studies, Quality of life

## Abstract

**Background:**

Although quality of life (QOL) improves over time for most breast cancer patients after their treatment, some patients may show different patterns of QOL. Beyond determining distinct QOL trajectories, identifying characteristics of patients who have different trajectories can help identify breast cancer patients who may benefit from intervention. We aimed to identify trajectories of QOL in breast cancer patients for one year after the end of primary treatment, to determine the factors influencing these changes.

**Methods:**

This longitudinal study recruited 140 breast cancer patients. Patients' QOL, symptom experience, self-efficacy, and social support were assessed using the Functional Assessment of Cancer Therapy Scale-G, Memorial Symptom Assessment Scale-Short Form, Self-Efficacy Scale for Self-Management of Breast Cancer, and Interpersonal Support Evaluation List-12. Data were collected immediately after the end of primary treatment (T1) and at three (T2), six (T3), and 12 months (T4) after primary treatment. Group-based trajectory modeling was used to identify distinct subgroups of patients with similar patterns of QOL change after treatment. A one-way analysis of variance was used to determine which variables were associated with trajectory membership. A multinomial logistic regression was performed to identify factors associated with trajectory group membership.

**Results:**

We analyzed 124 patients (mean age: 48.75 years). Latent class analysis of the QOL identified three trajectory groups: the low QOL group (*n* = 27; 21.1%), moderate QOL group (*n* = 57; 45.3%), and high QOL group (*n* = 40; 33.6%). The low QOL group showed consistently low QOL after the end of primary treatment, and the moderate QOL group showed a slight decrease in QOL from T1 to T3, which returned to the T1 level at T4. The high QOL group maintained a consistently high QOL. By multinomial logistic regression, psychological symptoms (odds ratio [OR] 0.46, 95% confidence interval [CI] 0.22–0.99) predicted a moderate QOL, and both psychological symptoms (OR 0.19, 95% CI 0.07–0.51) and belonging support (OR 1.60, 95% CI 1.06–2.39) predicted a high QOL.

**Conclusion:**

Identifying high-risk groups for reduced QOL after the end of primary treatment is necessary. Moreover, psychosocial interventions should be provided to alleviate psychological symptoms and increase belonging support to enhance patients' QOL.

*Trial registration* Not registered.

## Background

Breast cancer is increasing worldwide, ranking second among cancers occurring in women in Korea [1]. However, the survival rate of breast cancer patients has been increasing with the development of screening and adjuvant therapies in the past few decades [2]. Moreover, while the incidence of breast cancer is high in women aged 50 or older in foreign countries, in Korea, breast cancer is diagnosed most frequently in women in their 40 s (39.9%), which means that these women live for a longer time as breast cancer survivors after the completion of treatment [1].

The successful completion of adjuvant treatment after surgery is the beginning of the transition from cancer patient to cancer survivor [3, 4]. After primary treatment has been completed, patients with breast cancer can generally stay healthy and return to their former live [4]. However, during the transition period, after primary treatment ends, breast cancer patients may experience varying degrees of long-term physical, social, and psychological distress, complicating their survivorship with significant challenges [3]. Although some cancer-related concerns tend to decrease with time, many breast cancer patients face physical (physical activity, fatigue, pain, sleep disturbance), psychological (anxiety, depression, fear, low self-confidence), and social (avoidance, re-employment) problems related to the sequelae of treatment [5, 6]. These problems reduce adaptability and quality of life (QOL) in cancer survivors who have to learn and adapt to "Living with Cancer," and pose a significant challenge in the recovery process [3, 5].

To identify the duration and components of an intervention program that supports the transition from cancer patients to survivors, empirical evidence needs to be accumulated through an integrated investigation of changes in the QOL of breast cancer patients and the factors affecting these changes [6, 7]. In previous studies, factors affecting the QOL of breast cancer survivors included physical and psychological symptoms, self-efficacy [7–9], and social support [10–12]. The greater the symptoms experienced by breast cancer patients, the higher the psychological distress level, the lower their physical and social functioning, and the worse the overall QOL [13]. Self-efficacy positively affects the QOL of breast cancer survivors by helping them actively cope with cancer-related health problems [9] and maintaining self-management and health behavior for various symptoms [14]. Social support is also a representative environmental factor affecting the QOL of breast cancer patients; the provision of sufficient social support, such as feeling protected or receiving help from others [15, 16], assists survivors in actively coping with health problems [17] and finding positive meaning in life [18], which ultimately improves their QOL [10, 12, 16].

However, most studies have focused only on the treatment period [6], and studies on cancer survivors have been cross-sectional [7, 19]; thus, there is a lack of evidence on the changes in QOL in breast cancer patients and the factors affecting these changes during the transition period from being cancer patients to survivors. Longitudinal study and trajectory analysis facilitate individualized patient access. Individual management based on this approach can be used as a guide to determine realistic intervention directions. Moreover, identifying baseline determinants of trajectory patterns of QOL over time, defined as a baseline tracking model, helps develop timely interventions to improve QOL later [20]. Therefore, this longitudinal study aimed to identify QOL trajectory patterns of breast cancer patients during the first year after the end of primary treatment and the baseline determinants influencing these changes.

## Research questions

- What are the QOL trajectory patterns of breast cancer patients during the first year after the end of primary treatment?
- What determinants at baseline influence QOL trajectory patterns of breast cancer patients during the first year after the end of primary treatment?

## Methods

### Study design and participants

This was a longitudinal study to investigate the QOL trajectory patterns of breast cancer patients for 12 months from the end of primary treatment. In total, 140 breast cancer patients at a University hospital, who underwent a mastectomy and had completed adjuvant therapy such as chemotherapy or radiotherapy not more than a month previously, were selected through random sampling. The inclusion criteria were as follows: adults (1) aged 19 to 64 years, (2) with stage 1, 2, or 3 breast cancer who had completed chemotherapy or radiotherapy following surgery, (3) had no recurrence or metastasis, and (4) could communicate in Korean and fill out questionnaires. Patients receiving hormone therapy or targeted therapy to prevent recurrence were included in the study. The exclusion criteria were patients (1) diagnosed with a psychiatric disorder, such as adjustment disorder, obsessive–compulsive disorder, or anxiety disorder, or taking related drugs, and (2) those with health problems that might have caused cognitive impairment, such as stroke or dementia. Using the patient list registered at the hospital, from April to August 2018, breast cancer patients who met the selection criteria and gave their written consent to participate in the study were selected. Data were collected at four time points: immediately after the end of primary treatment (T1) and at 3 months (T2), 6 months (T3), and 12 months (T4) after the end of primary treatment.

On calculating with a significance level of 0.05, an odds ratio of 2.0 [7], and a power of 0.80 using G power analysis [21], the number of participants required was 113; therefore, 140 patients were recruited, considering a dropout rate of approximately 20%. Sixteen patients were lost to follow-up; 124 patients were included in the final analysis.

### Study instrument

#### Quality of life

Quality of life was measured using 27 items of the Korean version of the Functional Assessment of Cancer Therapy Scale-G (FACT-G) developed by the Functional Assessment of Chronic Illness Therapy Measurement System (FACIT). This scale assesses physical (7 items), social/family (7 items), emotional (6 items), and functional well-being (7 items) over the previous week; responses for each item range from 0 points for "strongly disagree" to 4 points for "strongly agree," and the higher the score, the higher the QOL. The validity and reliability of the Korean version of the FACT-G [22] had a Cronbach's ɑ of 0.78 − 0.90, while Cronbach's ɑ in this study was 0.85 − 0.91.

#### Symptom experience

Symptom experience was measured using the Memorial Symptom Assessment Scale-Short Form (MSAS-SF), in which patients rate symptom distress associated with 28 physical and four psychological symptoms [23]. Physical symptoms are included with two written description items and rated on a Likert scale (not at all: 0.8; a little bit: 1.6; somewhat: 2.4; quite a bit: 3.2; and very much: 4.0). Psychological symptoms are a total of four items rated as 0 points for "rarely" and 4 points for "almost constantly." The scores are compared using the mean of each item, and the higher the score, the more severe the symptom distress. At the time of development, this scale had Cronbach's α of 0.80 [23], while in this study, Cronbach's α was 0.80.

#### Self-efficacy

In this study, self-efficacy was measured using the Self-Efficacy Scale for Self-Management of Breast Cancer (SESSM-B), developed by Lee et al. [14]. This tool consists of 13 items, including coping with psycho-informational demand (3 items), maintenance of a healthy lifestyle (3 items), management of side effects (3 items), therapeutic compliance (2 items), and sexual life (2 items). The items were rated on a Likert scale ranging from 1 (strongly disagree) to 5 (strongly agree). The higher the sum of the scores, the higher the self-efficacy for self-management. The scale's reliability at the time of development was Cronbach's α = 0.78 [14], and the reliability in this study was Cronbach's α = 0.80.

#### Social support

Social support was assessed using the Korean version of the Interpersonal Support Evaluation List-12 (ISEL-12) [15] developed by Cohen [24]. This tool comprises 12 questions, with four response options: "not true at all," “not true,” “true,” and “very true” is to be selected. The ISEL-12 yields a total score that describes overall perceived social support and three subscales representing perceived availability of appraisal (advice or guidance), belonging (empathy, acceptance, concern), and tangible (help or assistance, such as material or financial aid) social support [24]. The higher the score, the higher the level of social support. The reliability of the Korean version of the ISEL-12 at the time of development was Cronbach's α = 0.87 [15], and the reliability in this study was Cronbach's α = 0.87.

### Data collection

Participants were recruited from May to August 2018. The post-investigation and follow-up were conducted face-to-face in the outpatient clinic when the patients visited the hospital for further management; otherwise, follow-up was conducted by mail. Clinical data were extracted from the patients' medical records using a data collection sheet. The data were processed by assigning a unique number to each individual in accordance with the Privacy Policy. Each time data was collected, a reward was provided to the patients who participated in the study.

### Data analysis

Descriptive statistics were used to summarize all participants' sociodemographic and disease-related characteristics and major study variables. Normality assumptions of the dependent variable was checked using the Kolmogorov–Smirnov test. Group-based trajectory modeling was used to identify different patterns of the overall QOL trajectory over time. The SAS 9.4 software (SAS Institute, Inc., Cary, NC) was used to estimate the model and calculate model performance indexes for alternative models based on the Bayesian information criterion (BIC) [25]. Low BIC values are interpreted as a good model fit to the data when an additional latent class is included [26]. Additionally, a model with more than 10% of the sample was selected for the minimum number of participants in a trajectory group. A value of 0.7 or higher was selected as the probability of a participant belonging to a specific trajectory group [27].

The Chi-square test was conducted to examine differences in participants’ sociodemographic and disease-related characteristics according to trajectory membership. Analysis of variance was conducted for differences in symptom experience, social support, and self-efficacy, and post hoc analysis was performed using Scheffé’s multiple comparison analysis. A multinomial logistic regression (MLR) was performed to identify the factors influencing the changes in patients’ QOL by introducing the variables that showed statistically significant univariate analysis variables, including perceived economic burden, symptom experience, self-efficacy, and social support. The MLRs results were reported as odds ratios (ORs) and 95% confidence intervals (95% CIs).

### Ethical considerations

This study was conducted with the approval of the Institutional Review Board of Ajou University Hospital (AJIRB-SBR-SUR-18–122). The study was conducted according to the principles expressed in the Declaration of Helsinki. Prior to the data collection, a research assistant explained the purpose and content of the study, study ethics, and data collection methods when the patients visited the outpatient clinic. The survey was conducted only with patients who understood the study process and provided written consent to participate.

## Results

### Participants’ characteristics and major study variables

A total of 124 breast cancer patients participated in the study. The mean age of the participants was 48.75 years (± 8.01). The number of participants who were high school graduates or had lower educational qualifications was 72 (58.0%), 111 (89.5%) participants had reported that they had a spouse, and 84 (67.7%) participants had little economic burden from cancer treatment. Seventy-two (58.1%) participants were diagnosed with stage 1 cancer. Ninety-six (77.4%) participants underwent partial mastectomy, and 70 (56.5%) were treated with chemotherapy, 113 (91.1%) with radiation therapy, and 108 (87.1%) with hormone therapy, as described in Table 1.

**Table 1:** Comparisons of sociodemographic and disease-related characteristics among three QOL trajectory patterns (*N* = 124)

Characteristics	Categories	Total (*N* = 124)	Consistently low QOL group (*N* = 27)	Consistently moderate QOL group (*N* = 57)	Consistently high QOL group (*N* = 40)	χ^2^ or F	*P*
		*N* (%) or Mean ± SD	*N* (%) or Mean ± SD	*N* (%) or Mean ± SD	*N* (%) or Mean ± SD		
Age (years, range: 26–54)		48.75 ± 8.01	49.70 ± 6.63	49.37 ± 7.99	47.23 ± 8.82	1.09	0.340
	~ 44	39 (31.5)	7 (5.6)	17 (13.7)	15 (12.1)		
	46 ~ 54	51 (41.1)	13 (10.5)	24 (19.4)	14 (11.3)		
	55 ~	34 (27.4)	7 (5.6)	16 (12.9)	11 (8.9)		
Educational level	≤ High school	72 (58.0)	16 (12.9)	37 (29.9)	19 (15.3)	2.95	0.229
	≥ University	52 (42.0)	11 (8.9)	20 (16.1)	21 (16.9)		
Spouse	No	13 (10.5)	4 (3.2)	2 (1.6)	7 (5.6)	5.59	0.061
	Yes	111 (89.5)	23 (18.6)	55 (44.4)	33 (26.6)		
Religion	No	67 (54.0)	11 (8.9)	33 (26.6)	23 (18.5)	2.46	0.293
	Yes	57 (46.0)	16 (12.9)	24 (19.4)	17 (13.7)		
Perceived economic status	High	7 (5.6)	3 (2.4)	2 (1.6)	2 (1.6)	10.21	0.037
	Middle	95 (76.6)	15 (12.1)	45 (36.3)	35 (28.2)		
	Low	22 (17.8)	9 (7.3)	10 (8.1)	3 (2.4)		
Perceived economic burden	None	28 (22.6)	4 (3.2)	6 (4.8)	18 (14.5)	20.33	< 0.001
	Little	84 (67.7)	18 (14.5)	47 (37.9)	19 (15.3)		
	Severe	12 (9.7)	5 (4.0)	4 (3.2)	3 (2.4)		
Employment status	Unemployed	83 (66.9)	18 (14.5)	38 (30.6)	27 (21.8)	0.01	0.996
	Employed	41 (33.1)	9 (7.3)	19 (15.3)	13 (10.5)		
Stage of cancer	1	72 (58.1)	16 (12.9)	32 (25.8)	24 (19.4)	2.19	0.700
	2	38 (30.6)	9 (7.3)	16 (12.9)	13 (10.5)		
	3	14 (11.3)	2 (1.6)	9 (7.3)	3 (2.4)		
Type of surgery	Partial mastectomy	96 (77.4)	22 (17.7)	42 (33.9)	32 (25.8)	0.86	0.650
	Total mastectomy	28 (22.6)	5 (4.0)	15 (12.1)	8 (6.5)		
Chemotherapy	No	54 (43.5)	11 (8.9)	25 (20.2)	18 (14.5)	2.15	0.905
	Yes	70 (56.5)	16 (12.9)	32 (25.8)	22 (17.7)		
Radiotherapy	No	11 (8.9)	3 (2.4)	6 (4.8)	2 (1.6)	1.10	0.576
	Yes	113 (91.1)	24 (19.4)	51 (41.1)	38 (30.6)		
Hormone therapy	No	16 (12.9)	3 (2.4)	6 (4.8)	7 (5.6)	1.12	0.572
	Yes	108 (87.1)	24 (19.4)	51 (41.1)	33 (26.6)		
Target therapy	No	104 (83.9)	22 (17.7)	52 (41.9)	30 (24.2)	4.72	0.094
	Yes	20 (16.1)	5 (4.0)	5 (4.0)	10 (8.1)		

*N* number, *SD* standard deviation, *QOL* quality of life

Of the total group, the mean score for physical symptoms was 0.99 (± 0.68), and that for psychological symptoms was 1.35 (± 1.02). The mean self-efficacy score was 49.48 (± 8.22), and the mean social support score was 37.49 (± 6.43). The study participants scored 12.77 (± 2.56) on appraisal support, 11.95 (± 2.47) on tangible support, and 12.77 (± 2.40) on belonging support (Table 2).

**Table 2:** Comparison of symptom experience, self-efficacy, and social support at baseline among QOL trajectory patterns (*N* = 124)

Variables	Total (*N* = 124)	Consistently low QOL group (Group 1) (*N* = 27)	Consistently moderate QOL group (Group 2) (*N* = 57)	Consistently high QOL group (Group 3) (*N* = 40)	F	p*	*Scheffe* *post-hoc test*
	Mean ± SD	Mean ± SD	Mean ± SD	Mean ± SD			
*Symptom experience*							
Physical symptoms	0.99 ± 0.68	1.46 ± 0.70	1.00 ± 0.60	0.65 ± 0.60	13.81	< 0.001	Group 1 > Group 2 > Group 3
Psychological symptoms	1.35 ± 1.02	2.20 ± 0.94	1.38 ± 0.91	0.74 ± 0.78	22.44	< 0.001	Group 1 > Group 2 > Group 3
*Self-efficacy*	49.48 ± 8.22	46.15 ± 8.36	49.28 ± 8.33	52.00 ± 7.25	4.34	0.015	Group 1 < Group 3
*Social support*	37.49 ± 6.43	33.26 ± 7.63	37.40 ± 5.74	40.48 ± 4.76	11.98	< 0.001	Group 1 < Group 2 < Group 3
Appraisal support	12.77 ± 2.56	11.48 ± 2.99	12.67 ± 2.44	13.78 ± 2.01	7.19	0.001	Group 1 < Group 3
Tangible support	11.95 ± 2.47	10.48 ± 2.85	12.07 ± 2.31	12.78 ± 1.99	7.84	0.001	Group 1 < Group 2, Group 1 < Group 3
Belonging support	12.77 ± 2.40	11.30 ± 2.63	12.67 ± 2.17	13.93 ± 1.98	11.45	< 0.001	Group 1 < Group 2 < Group 3

*N* number, *SD* standard deviation, *QOL* quality of life  *One way ANOVA test

### Identifying QOL trajectory patterns

**Fig. 1:**
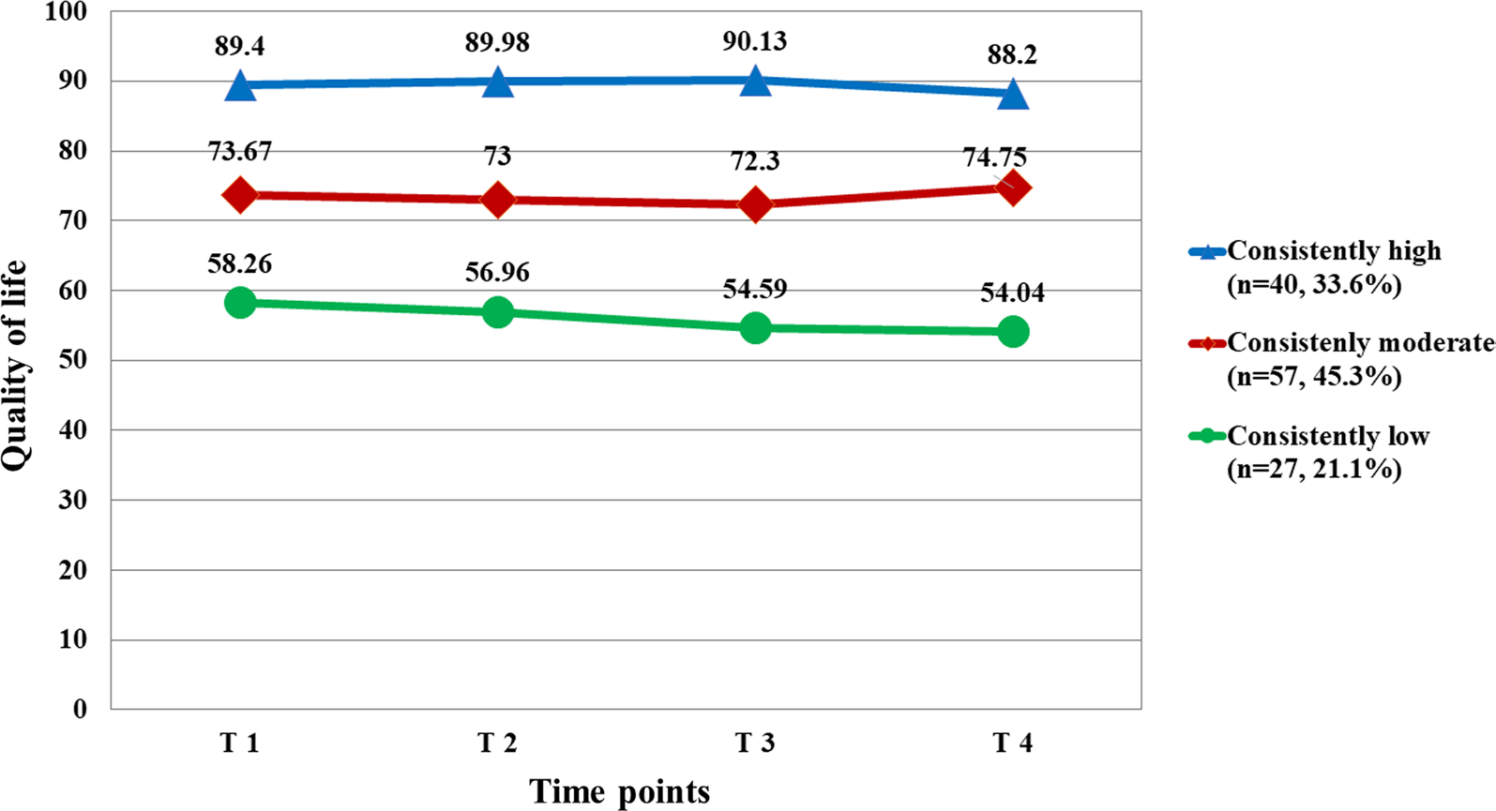
Predicted and observed quality of life for each trajectory group

As a result of the trajectory analysis, three trajectories representing changes in QOL were identified (Fig. 1). The BIC value of the three trajectory groups was -1964.08, and the number of participants in each group was a minimum of 27 (Group 1), which was more than 10% of the sample (Table 3). According to the characteristics of distribution, the three QOL trajectory patterns were named “consistently good” (*n* = 40, 33.6%), “consistently moderate” (*n* = 57, 45.3%), and “consistently low” (*n* = 27, 21.1%). The means of QOL in three trajectory patterns across four time points are shown in Fig. 1. As shown, the means of QOL at five time points for the “consistently good” QOL trajectory pattern were stable and all higher than those of all participants. Means of QOL at five time points for the participants of “consistently moderate” QOL trajectory patterns were stable and slightly smaller than those of all participants. For the “consistently low” QOL trajectory pattern, QOL substantially declined for one year since the end of primary treatment (T4) and did not recover to the T1 level even after a year.

**Table 3:** Model selection results for QOL for 124 women with breast cancer

No. of groups	BIC	Estimated probability (% in each group)				
		1	2	3	4	5
1	− 2067.21	100.0				
2	− 1980.06	42.6	57.4			
3	− 1964.08	21.1	45.3	33.6		
4	− 1946.90	4.1	33.3	37.4	25.2	
5	− 1946.19	4.1	25.6	7.0	37.7	25.5

*BIC* Bayesian Information Criteria, *QOL* quality of life

### Association between possible predictors and trajectory groups for overall QOL

Table 1 shows the comparisons of sociodemographic and disease-related characteristics at baseline on three QOL trajectory patterns. As a result, only perceived economic burden due to cancer treatment (χ^2^ = 20.33, *p* < 0.001) showed a statistically significant difference depending on the groups. Next, differences in mean scores for the physical and psychological symptoms, self-efficacy, and social support at baseline were analyzed for each QOL group (Table 2). Results showed statistically significant differences in physical symptoms (F = 13.81, *p* < 0.001), psychological symptoms (F = 22.44, *p* < 0.001), self-efficacy (F = 4.34, *p* = 0.015), appraisal support (F = 7.19, *p* = 0.001), tangible support (F = 7.84, *p* = 0.001), and belonging support (F = 11.45, *p* < 0.001).

### Identifying important determinants of QOL trajectory patterns

After the latent groups were identified, MLRs were conducted using the consistently low QOL group as the reference for perceived economic burden, symptom experience, self-efficacy, and social support. This yielded comparisons of the consistently high and consistently low QOL groups and the consistently moderate and consistently low QOL groups (Table 4).

**Table 4:** Determinants of baseline of quality of life trajectory patterns (Consistently low QOL as the referent) (*N* = 124)

Variables	Consistently moderate QOL group					Consistently high QOL group				
	β	*p*	Odds ratio	95% confidence intervals		β	*p*	Odds ratio	95% confidence intervals	
*Symptom experience*										
Physical symptoms	− 0.42	0.380	0.66	0.26	1.68	− 0.77	0.227	0.46	0.13	1.62
Psychological symptoms	− 0.77	0.046	0.46	0.22	0.99	− 1.64	0.001	0.19	0.07	0.51
*Social support*										
Appraisal support	− 0.15	0.403	0.86	0.62	1.22	0.00	0.998	1.00	0.66	1.51
Tangible support	0.18	0.257	1.19	0.88	1.62	− 0.01	0.973	0.99	0.70	1.41
Belonging support	0.16	0.315	1.17	0.86	1.59	0.47	0.024	1.60	1.06	2.39
Self-efficacy	0.02	0.600	1.02	0.95	1.10	0.02	0.644	1.02	0.93	1.12
*Perceived economic burden (ref: none)*										
Little	0.02	0.600	1.02	0.95	1.10	0.02	0.644	1.02	0.93	1.12
Severe	− 0.10	0.926	0.91	0.11	7.45	0.77	0.529	2.16	0.20	23.79
Constant	− 1.19	0.579	0.66	0.26	1.68	− 3.58	0.227			

− 2Log Likelihood = 190.82, χ^2^ (*df*) = 70.62 (16), *p* < 0.001

*QOL* quality of life

In the consistently high QOL group versus consistently low QOL group comparison, psychological symptoms and belonging support significantly differentiated the two groups (*p* < 0.05; Table 4). The odds ratio for psychological symptom scores at T1 was 0.19 (95% CI 0.07-0.51), indicating that as a patient’s score increased by 1 point at T1, the patient had 5.26 times higher odds of belonging to the low QOL group than the consistently high QOL group (*p* = 0.001). Furthermore, the odds ratio for belonging support at T1 was 1.60 (95% CI 1.06–2.39), indicating that a patient had 1.60 times higher odds of being in the consistently high QOL group compared with the consistently low QOL, with an increase of 1 point in belonging support at T1 (*p* = 0.024).

In the consistently moderate QOL group versus consistently low QOL group comparison, only psychological symptoms at T1 significantly differentiated the two groups. The odds ratio for psychological symptom scores at T1 was 0.46 (95% CI 0.22–0.99, *p* = 0.046), indicating that as a patient’s score increased by 1 point at T1, the patient had 2.17 times higher odds of belonging to the low QOL group compared to the consistently moderate QOL group.

## Discussion

This study identified trajectory groups according to changes in the QOL of breast cancer survivors whose primary treatment was completed. Factors influencing the membership of different trajectory groups were also determined. Approximately 80% of the participants were classified into the consistently moderate or high QOL groups in this study. This is consistent with a previous study investigating the QOL in breast cancer survivors (*N* = 653) after completion of primary treatment [28], where approximately 70% of the participants were classified into the moderate or higher QOL groups. Participants in the consistently moderate QOL group (45.3%) showed a gradual decline in their QOL after the end of primary treatment. However, they recovered to the consistently moderate QOL group after completing treatment after 12 months. In addition, participants (33.6%) in the high QOL group maintained a high QOL for 12 months after the end of primary treatment.

Approximately 20% of the participants were classified into the low QOL group. Longitudinal studies that followed breast cancer survivors up to 12 months after the end of primary treatment reported improvements in QOL over time [9, 29]. However, this study found that, for 21.8% of patients, there was no improvement in QOL even after treatment. In this study, the consistently low QOL group had a mean QOL score of 15 to 30 points lower than the consistently moderate and high QOL groups at the end of primary treatment (T1), and the score continued to decrease during the follow-up period, resulting in a vast difference in scores compared with the other two groups. In particular, unlike the moderate and high QOL groups, the consistently low QOL group showed significantly decreased QOL scores six months after treatment (T3). In addition, some breast cancer patients feel vulnerable in terms of physical, psychological, social, and spiritual aspects throughout the lengthy recovery process, even after completing medical treatment [2]. Considering that the QOL one year after the end of primary treatment is essential in determining the QOL as a breast cancer survivor, strategies are needed to periodically identify QOL for high-risk groups using a standardized questionnaire during this period [30].

Furthermore, among the factors affecting the QOL trajectory patterns, patients’ psychological symptoms predicted a moderate QOL, and both psychological symptoms and belonging support predicted a high QOL. First, a patient with fewer psychological symptoms at the end of primary treatment is more likely to belong to the moderate or high quality of life groups. This is similar to a previous study that followed up 126 breast cancer patients before chemotherapy to 12 months after chemotherapy completion [30] and reported that the higher the psychological distress, such as depression and anxiety, the lower the QOL. Breast cancer patients may experience anxiety about health, misunderstanding of physical symptoms, anxiety, uncertainty about cancer recurrence, depression, and fatigue after treatment is completed [2, 31]. Psychological distress has a direct impact on the patient's ability to return to life or return to work [32], as well as negligent self-management to prevent recurrence [31]. It has been reported that approximately 20% of breast cancer survivors experience severe psychological distress even after treatment [33, 34]. Therefore, it is necessary to monitor the levels of psychological distress regularly and refer them to mental health experts, if necessary. In addition, since psychosocial intervention effectively alleviates psychological distress [35], it is desirable to include psychosocial interventions in the integrated treatment plan for cancer survivors, beginning at the end of primary treatment.

Second, this study showed that the higher the score for belonging support, the higher the likelihood of belonging to the consistently high QOL group. This is consistent with a cross-sectional study that reported that belonging support is a major factor influencing the QOL of breast cancer survivors (*N* = 150) whose primary treatment has ended [16]. A longitudinal study on 30 women with non-metastatic breast cancer also reported that emotional support at the time of diagnosis significantly affected the QOL and physical well-being of patients six months after the end of primary treatment [10]. This suggests that survivors who experience a sense of belonging to their families, friends, or healthcare professionals, whom they shared a close relationship with and received emotional support from, have a high QOL. This aspect of social support was found to have mediating and moderating effects on the relationship between psychological symptoms, such as depression, and the QOL of breast cancer survivors [16, 36]. Thus, to improve the QOL of breast cancer survivors, efforts should be made to maintain a sense of belonging with healthcare professionals by maintaining communication with survivors through various methods even after the end of treatment [37].

In this study, however, appraisal and tangible social support did not sufficiently explain the variance in QOL. Appraisal support refers to the provision of feedback regarding performance, or personal qualities, and tangible support is the provision of financial assistance, material goods, or services [24]. Previous studies suggested that some types of social support may negatively affect breast cancer patients' QOL [38]. However, most of the previous studies identified the relationship between overall social support and quality of life, so it is limited to comparing the results of this study. Future research is required to identify which types of social support are factors that affect changes in QOL.

This study's strengths include the application of a person-centered statistical approach that is flexible and adaptable in capturing between-individual differences among clusters of individuals with similar responses over time. In addition, high-risk groups that are likely to lower QOL were identified by identifying factors affecting the trajectory of QOL, focusing on variables measured at the end of cancer-related primary treatment. However, caution is required while interpreting the results due to the following limitations. Firstly, participants were only selected from a single medical center by convenience sampling and completed at least four repeated observations in Korea, which may limit the representativeness of samples in patients with breast cancer. Second, the sample size for each trajectory group was less than 25% of the sample, and the measurement time points were insufficient for nine months after the end of cancer treatment, which may have affected the trajectory shape [39]. Third, substantial change in HRQOL can be over- or under-estimated without adjusting for a response shift. Therefore, future research is needed to determine whether response shift implies an unwanted potential bias. Finally, this study evaluated the QOL in breast cancer patients using the FACT-G, which is a cancer patient-specific tool. This was to reduce the double measurement error for symptom experience when using the FACT-B, a QOL tool reflecting breast cancer-specific domains. However, there is a possibility that treatment-related effects (e.g., premature menopause and decline in cognitive function) were not sufficiently reflected in the QOL of breast cancer survivors. If the breast cancer specific QOL questionnaire such as FACT-B is used in the future, it will be possible to evaluate multifaceted QOL comprehensively.

## Conclusions

This study examined changes in the QOL of breast cancer patients for 12 months after the end of primary treatment and identified the factors influencing these changes. Most patients maintained or recovered a moderate or high QOL over time. However, approximately 20% of the participants showed a consistently low QOL at the end of primary treatment, with continued deterioration in QOL over time. Breast cancer patients who experienced a low level of psychological symptoms and high belonging support at the end of treatment were more likely to have a moderate or high QOL. Therefore, it is necessary to identify the high-risk group for low QOL by determining breast cancer patients’ symptom experience and social support level at the end of treatment. Moreover, it is necessary to provide psychosocial interventions to alleviate psychological symptoms and improve belonging support to enhance their QOL.

## Data Availability

All data generated or analyzed during this study are included in this published article.

## Abbreviations

95% CI: 95% Confidence interval
FACT-B: The Functional Assessment of Cancer Therapy Scale-B
FACT-G: The Functional Assessment of Cancer Therapy Scale-G
ISEL-12: The Interpersonal Support Evaluation List-12
MSAS-SF: The Memorial Symptom Assessment Scale-Short Form
MLR: Multinomial logistic regression
OR: Odds ratio
QOL: Quality of life
SESSM-B: The Self-Efficacy Scale for Self-Management of Breast Cancer

## References

[1] Korean breast cancer society. Breast cancer facts & figures. Korean Breast Cancer Society 2020. Ministry of Health and Welfare; 2020. http://www.kbcs.or.kr/sub02/sub04.html. Accessed 28 Feb 2022.

[2] Schapira L, Zheng Y, Gelber SI, Poorvu P, Ruddy KJ, Tamimi RM (2022). Trajectories of fear of cancer recurrence in young breast cancer survivors. Cancer.

[3] Garofalo JP, Choppala S, Hamann HA, Gjerde J (2009). Uncertainty during the transition from cancer patient to survivor. Cancer Nurs.

[4] Miller K, Merry B, Miller J (2008). Seasons of survivorship revisited. Cancer J.

[5] Pinheiro LC, Wheeler SB, Reeder-Hayes KE, Samuel CA, Olshan AF, Reeve BB (2017). Investigating associations between health-related quality of life and endocrine therapy underuse in women with early-stage breast cancer. J Oncol Pract.

[6] Wöckel A, Schwentner L, Krockenberger M, Kreienberg R, Janni W, Wischnewsky M (2017). Predictors of the course of quality of life during therapy in women with primary breast cancer. Qual Life Res.

[7] Xia J, Tang Z, Deng Q, Yang R, Wang J, Yu J (2018). Predictors of the quality of life in Chinese breast cancer survivors. Breast Cancer Res Treat.

[8] Lee BG, Lee TS, Kim SH (2019). Mediation effect of self-efficacy on the relationship between perceived self-management support and health-related quality of life among cancer survivors. J Korean Acad Nurs.

[9] Rottmann N, Dalton SO, Christensen J, Frederiksen K, Johansen C (2010). Self-efficacy, adjustment style and well-being in breast cancer patients: a longitudinal study. Qual Life Res.

[10] Lantheaume S, Fernandez L, Lantheaume S, Moták L, Conceição SB (2022). Quality of life in patients with non-metastatic breast cancer: evolution during follow-up and vulnerability factors. Support Care Cancer.

[11] DuMontier C, Clough-Gorr KM, Silliman RA, Stuck AE, Moser A (2018). Health-related quality of life in a predictive model for mortality in older breast cancer survivors. J Am Geriatr Soc.

[12] Leung J, Pachana NA, McLaughlin D (2014). Social support and health-related quality of life in women with breast cancer: a longitudinal study. Psychooncology.

[13] Browall M, Östlund U, Henoch I, Wengström Y (2013). The course of health related quality of life in postmenopausal women with breast cancer from breast surgery and up to five years post-treatment. Breast.

[14] Lee R, Kim SH, Lee KS, Seo MK (2012). Development and validation of self-efficacy scale for self-management of breast cancer (SESSM-B). J Korean Acad Nurs.

[15] Kim DH, Lee HK, Kim JW, Lee K (2012). Reliability and validity of the Korean version of interpersonal support evaluation list-12 (ISEL-12). J Korean Neuropsychiatr Assoc.

[16] Huang CY, Hsu MC (2013). Social support as a moderator between depressive symptoms and quality of life outcomes of breast cancer survivors. Eur J Oncol Nurs.

[17] Yang S, Kim E (2015). The relationship among the coping style, social support, and post-traumatic stress disorder in breast cancer patients treated with chemotherapy. Korean J Hospice Palliat Care.

[18] Kim YJ, Lee KJ (2010). Relationship of social support and meaning of life to suicidal thoughts in cancer patients. J Korean Acad Nurs.

[19] Ou HT, Chung WP, Su PF, Lin TH, Lin JY, Wen YC, Fang W (2019). Health-related quality of life associated with different cancer treatments in Chinese breast cancer survivors in Taiwan. Eur J Cancer Care.

[20] Lin KC, Yan CF, Cheng SF, Gau ML (2013). Evaluation of time-varying and cumulative effects in nursing in a longitudinal study. Nurs Res.

[21] Faul F, Erdfelder E, Lang AG, Buchner A (2007). G*Power 3: A flexible statistical power analysis program for the social, behavioral, and biomedical sciences. Behav Res Methods.

[22] Lee EH, Chun M, Kang S, Lee HJ (2004). Validation of the Functional Assessment of Cancer Therapy-General (FACT-G) scale for measuring the health-related quality of life in Korean women with breast cancer. Jpn J Clin Oncol.

[23] Chang VT, Hwang SS, Feuerman M, Kasimis BS, Thaler HT (2000). The memorial symptom assessment scale short form (MSAS-SF). Cancer.

[24] Cohen S, Mermelstein R, Kamarck T, Hoberman HM, Sarason IG, Sarason BR (1985). Measuring the functional components of social support. Social support: theory, research and applications.

[25] Nagin DS (1999). Analyzing developmental trajectories: a semiparametric, group-based approach. Psychol Methods.

[26] Jones BL, Nagin DS, Roeder K (2001). A SAS procedure based on mixture models for estimating developmental trajectories. Sociol Methods Res.

[27] Nagin DS (2005). Group-based modeling of development.

[28] Goyal NG, Levine BJ, Van Zee KJ, Naftalis E, Avis NE (2018). Trajectories of quality of life following breast cancer diagnosis. Breast Cancer Res Treat.

[29] Ganz PA, Kwan L, Stanton AL, Bower JE, Belin TR (2011). Physical and psychosocial recovery in the year after primary treatment of breast cancer. J Clin Oncol.

[30] Park JH, Jung YS, Kim JY, Jo Y, Bae SH (2020). Trajectories of health-related quality of life in breast cancer patients. Support Care Cancer.

[31] Runowicz CD, Leach CR, Henry NL, Henry KS, Mackey HT, Cowens-Alvarado RL (2016). American Cancer Society/American Society of Clinical Oncology breast cancer survivorship care guideline. CA Cancer J Clin.

[32] Kim SY, Kissane DW, Richardson G, Senior J, Morgan J, Gregory P (2022). The role of depression and other psychological factors in work ability among breast cancer survivors in Australia. Psychooncology.

[33] Park JH, Chun M, Jung YS, Bae SH (2017). Predictors of psychological distress trajectories in the first year after a breast cancer diagnosis. Asian Nurs Res.

[34] Bidstrup PE, Christensen J, Mertz BG, Rottmann N, Dalton SO, Johansen C (2015). Trajectories of distress, anxiety, and depression among women with breast cancer: looking beyond the mean. Acta Oncol.

[35] Setyowibowo H, Yudiana W, Hunfeld JAM, Iskandarsyah A, Passchier J, Arzomand H (2022). Psychoeducation for breast cancer: a systematic review and meta-analysis. Breast.

[36] Ban Y, Li M, Yu M, Wu H (2021). The effect of fear of progression on quality of life among breast cancer patients: the mediating role of social support. Health Qual Life Outcomes.

[37] Janz NK, Friese CR, Li Y, Graff JJ, Hamilton AS, Hawley ST (2014). Emotional well-being years post-treatment for breast cancer: prospective, multi-ethnic, and population-based analysis. J Cancer Surviv.

[38] Culbertson MG, Bennett K, Kelly CM, Sharp L, Cahir C (2020). The psychosocial determinants of quality of life in breast cancer survivors: a scoping review. BMC Cancer.

[39] Gonzalez BD, Manne SL, Stapleton J, Myers-Virtue S, Ozga M, Kissane D (2017). Quality of life trajectories after diagnosis of gynecologic cancer: a theoretically based approach. Support Care Cancer.

